# Correspondence

**Published:** 1859-12

**Authors:** 


### Correspondence.

Messrs. Editors:—Permit me to say to you and to the profession, through you, a few words respecting our Conventions, both past and prospective; and first, I would remark that I altogether approve of the spirit of large Democratic Republicanism which prompted and guided the founders of the institution, in its formation, if that may be called an "institution" which acknowledges no written law or creed. Furthermore, I claim to sympathize with the feelings of kindness and liberality that have thus far characterized the meetings of the Convention, and in some degree to participate in the sentiments of generous rivalry that should stimulate those who are thus associated, to aspire to something worthy the favorable regard of their professional brethren and the public generally. The perennial fountain of information that has its sources in the observation and experience of the whole profession is not the least or last of its advantages; but while these and many other inducements are tendered to all who choose to participate in the meetings of the Convention, still there are some small annoyances, that would scarcely merit attention, only that like corruption in politics, like disease in the vital organs, or decay in the dental organs, or weeds in agricultural operations, they exhibit a tendency to increase, overgrow and monopolize every thing valuable, and consequently, the sooner they can be put down, the better.

One of the annoyances to which I refer is the indomitable propensity that some members manifest to speak on every topic that is up for discussion. Sometimes we hear remarks that many of us have heard repeated from the same lips annually, time out of mind; sometimes these remarks may appear pertinent enough to those who chance to hear them for the first time, but too often they appear more like a rehash from some old lecture originally prepared for the instruction of a class of dental students, who were waiting to be initiated into the mysteries of the science. Some evade the rule restricting members to one speech, by taking advantage of parliamentary usage to rise to explain, or ask an explanation, to repeat something that they had already said, and supposed, of course, the audience had forgotten, or to ask some one to repeat something that they themselves had heard and forgotten within the last half hour. It would, no doubt, appear to a disinterested spectator as if their only aim was to occupy the time, distract the attention, and embarrass the labors of the Convention.

Messrs. Editors, I lay claim to a fair share of charity towards the infirmities of my unfortunate fellow-creatures, when I see those infirmities accompanied with a moderate proportion of modesty; but when I see individuals obtrude their stupidity unnecessarily on those who can hear and understand and remember, I can not but regard it as decidedly annoying. It is generally thought to be a very disagreeable trick in a youth of tender years and small stature to require that every thing that he may chance to hear pass between older or more intelligent persons than himself, be repeated for his individual benefit; but when by a long course of indulgence, this grows into a confirmed habit, and in the mean time the boy grows to be six feet high and lubberly in proportion, the flagrancy and magnitude of the offense increase, in full proportion, with the size of the offender.

In conclusion, permit me to suggest as a remedy for these grievances, that hereafter the members take their seats in order as they subscribe their names and pay their dues, and instead of discussing the various branches of dental theory and practice, in the order in which they naturally occur, let each member be called on in regular order for whatever he may have of interest to the profession, reports of rare or difficult cases, with treatment, success or failure, with inferences, suggestions, etc., limiting to 10 or 15 minutes, and securing from perplexing question or other interruption; new modes of operation, materials or appliances; new theories of diseased or healthy function; even "the moulting of the temporary teeth by atrophy" might be introduced at the risk of the publisher. After each one has had opportunity to say what is uppermost in his mind, devote a session or two to the discussion of such topics as may be thought worthy of special consideration, permitting each member to speak in regular order, without interruption or catechization. There is ample opportunity outside of the Convention to advertise such inventions as are offered for sale to the profession, especially when the profits arising from the sale of such inventions are secured to their authors, under the sanction of the Patent Laws.

If the difficulties to which I have alluded can not be avoided in future, I for one, venture to predict that the enterprise must ultimately prove a failure. OLD RASP.

